# The Amino-terminal Domain of the Androgen Receptor Co-opts Extracellular Signal-regulated Kinase (ERK) Docking Sites in ELK1 Protein to Induce Sustained Gene Activation That Supports Prostate Cancer Cell Growth*[Fn FN2]

**DOI:** 10.1074/jbc.M116.745596

**Published:** 2016-10-28

**Authors:** Rayna Rosati, Mugdha Patki, Venkatesh Chari, Selvakumar Dakshnamurthy, Thomas McFall, Janice Saxton, Benjamin L. Kidder, Peter E. Shaw, Manohar Ratnam

**Affiliations:** From the ‡ Barbara Ann Karmanos Cancer Institute and Department of Oncology,; § Wayne State University School of Medicine, Detroit, Michigan 48201-2013 and; the ¶ School of Biomedical Sciences, University of Nottingham, Queen's Medical Centre, Nottingham NG7 2UH, United Kingdom

**Keywords:** androgen receptor, ETS transcription factor family, extracellular signal-regulated kinase (ERK), prostate cancer, transcription

## Abstract

The ETS domain transcription factor ELK1 is in a repressive association with growth genes and is transiently activated through phosphorylation by ERK1/2. In prostate cancer (PCa) cells the androgen receptor (AR) is recruited by ELK1, via its amino-terminal domain (A/B), as a transcriptional co-activator, without ELK1 hyper-phosphorylation. Here we elucidate the structural basis of the interaction of AR with ELK1. The ELK1 polypeptide motifs required for co-activation by AR *versus* those required for activation of ELK1 by ERK were systematically mapped using a mammalian two-hybrid system and confirmed using a co-immunoprecipitation assay. The mapping precisely identified the two ERK-docking sites in ELK1, the D-box and the DEF (docking site for ERK, F*X*FP) motif, as the essential motifs for its cooperation with AR(A/B) or WTAR. In contrast, the transactivation domain in ELK1 was only required for activation by ERK. ELK1-mediated transcriptional activity of AR(A/B) was optimal in the absence of ELK1 binding partners, ERK1/2 and serum-response factor. Purified ELK1 and AR bound with a dissociation constant of 1.9 × 10^−8^ m. A purified mutant ELK1 in which the D-box and DEF motifs were disrupted did not bind AR. An ELK1 mutant with deletion of the D-box region had a dominant-negative effect on androgen-dependent growth of PCa cells that were insensitive to MEK inhibition. This novel mechanism in which a nuclear receptor impinges on a signaling pathway by co-opting protein kinase docking sites to constitutively activate growth genes could enable rational design of a new class of targeted drug interventions.

## Introduction

The androgen receptor (AR)^2^ and other members of the nuclear receptor (NR) superfamily mediate the transcriptional activities of their ligands as well as some of their non-genomic actions (1–5). NRs in the cytosol, in the nucleus, or in association with plasma membrane proteins are known to interact with a variety of signaling pathway proteins, either as protein kinase substrates or as regulators of transcription or signal transduction. Although some of the client proteins of NRs in these pathways have been identified, including those shared by different NRs, there is a paucity of information on the structural elements that enable the mutual recognition of NRs and signaling proteins in both normal physiology and in pathogenesis.

Most early stage and advanced prostate tumors depend on AR for growth (6–11). The human AR is a 919-amino acid polypeptide with a basic structural organization typical of NRs (12). In the classical model of gene regulation by AR, ligand binding is critical for several events, including release of AR from a cytosolic chaperone complex as well as phosphorylation, stabilization, dimerization, and nuclear import of AR (12–14). Ligand binding is also needed for optimal binding of AR to DNA at well characterized response elements associated with target genes (15, 16). However, advanced PCa cells may acquire the ability to localize adequate AR to the nucleus where it is transcriptionally active through mechanisms that include AR amplification, hormone-independent phosphorylation of AR through hyper-activated signaling pathways, or overexpression of ligand-independent AR splice variants (17–20). Splice variants of AR have carboxyl-terminal truncations that lack the ligand binding domain (21). The above cellular changes in advanced PCa cells, as well as intratumoral androgen biosynthesis, could render the tumors resistant to conventional AR-targeted therapies, including surgical or chemical castration and androgen antagonists (22–27). Normal and malignant prostate epithelial cells appear to redirect androgen/AR signaling to regulate different sets of genes via tethering proteins that bind AR to chromatin (16, 28–31). Therefore, developing drugs that disrupt interactions of AR with a tethering protein required exclusively for growth is an attractive approach for overcoming resistance to current AR-targeted therapies and would also obviate the need for androgen ablation.

We have previously reported that the ETS family transcription factor ELK1 is essential for growth in PCa cells that are either dependent on androgen or independent of hormone but still dependent on the AR (31). In contrast, ELK1 was not required for growth in AR-negative PCa cells. In PCa cells, ELK1 is required wholly or in part for activation by androgen/AR of ∼27% of target genes, and these genes are enriched for clusters supporting cell cycle progression and mitosis (31). Promoter activation analyses, mammalian two-hybrid assays, and chromatin immunoprecipitation studies have indicated that ELK1 recruits AR as a transcriptional co-activator (31). Other investigators have extended these studies to demonstrate that ELK1 is similarly required for androgen-dependent growth of bladder cancer (32, 33). Moreover, chromatin sites of AR binding are highly enriched for ELK1 binding DNA cis-elements (34).

ELK1 belongs to the ternary complex factor subfamily of ETS proteins that characteristically bind to purine-rich GGA core sequences (35). ELK1 is activated through hyper-phosphorylation by ERK to transiently activate immediate early genes in association with the serum-response factor (SRF) (35–39). Accordingly, ELK1 is one of many substrates of ERK1/2 that have variable types and combinations of recognition motifs for ERK (40–42). In the absence of hyper-phosphorylation, ELK1 is in a repressive association with many genes (43). However, ELK1 is expressed in the clinical spectrum of prostate tumors (44), and in PCa cells the activation of AR target growth genes through ELK1 was constitutive and did not entail hyper-phosphorylation or activation of immediate early genes (31). In this study, we now elucidate the physical basis for the interaction between ELK1 and AR in the context of growth dependence of PCa cells on ELK1.

## Results

### 

#### 

##### Role of the Amino-terminal A/B Domain of AR in Functional Interactions with ELK1

**FIGURE 1.**
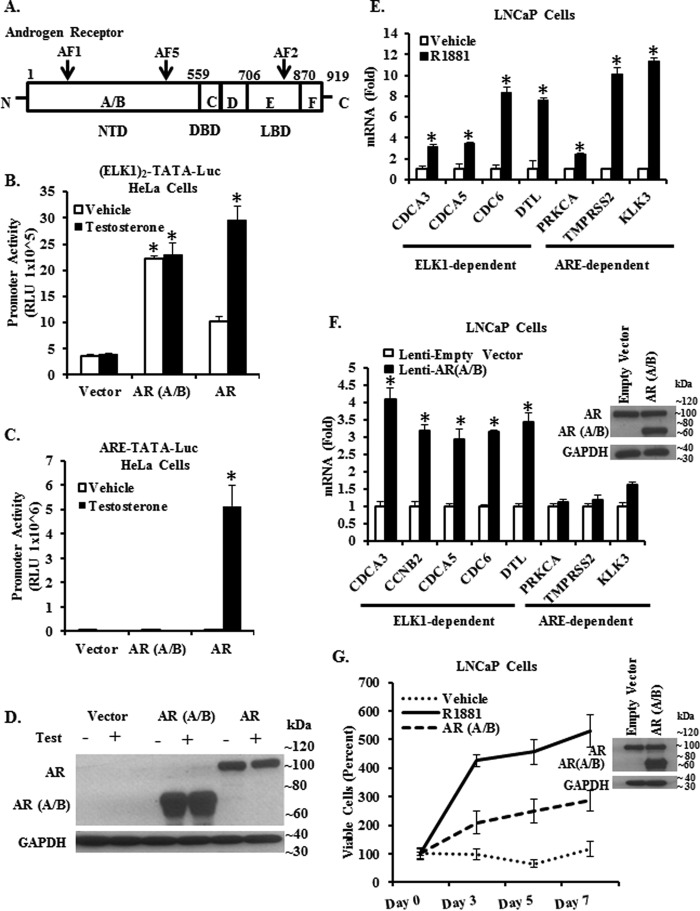
**Adequacy of the A/B domain of AR for functional interactions of AR with ELK1.** *A* shows a schematic for the organization of functional domains in AR. The A/B domain is the amino-terminal domain (*NTD*), which contains the ligand-independent activation functions AF1 and AF5. The C domain comprises the DNA binding domain (*DBD*) adjacent to a hinge region (*D*). The E domain encompasses the ligand binding domain (*LBD*) and the ligand-dependent activation function, AF2. The F domain represents the carboxyl-terminal domain. *B* and *C*, hormone-depleted HeLa cells were transfected with an ELK1-driven minimal promoter-luciferase reporter ((*ELK1*)_2_-*TATA-LUC*) (*B*) or with an androgen-response element-driven minimal promoter-luciferase reporter (ARE-TATA-Luc) (*C*) and co-transfected with expression plasmids for the AR A/B domain, full-length AR, or the plasmid vector control. The cells were treated with either testosterone (10 nm) or vehicle at the time of transfection. Luciferase activity was measured in the cell lysates 48 h post-transfection. *D* shows a Western blot of lysates from cells transfected with expression plasmids for either the full-length AR or the AR A/B domain and treated with either testosterone (10 nm) or vehicle for 48 h and probed using an antibody to the amino-terminal domain of AR or with antibody to GAPDH (loading control). *E,* hormone-depleted LNCaP cells were treated with R1881 (1 nm) or vehicle for 48 h. Total RNA from the cells was used to quantify mRNA levels for the indicated genes that were known to be either ELK1-dependent or ARE-dependent for activation by AR. *F,* hormone-depleted LNCaP cells transduced using lentivirus expressing either the AR A/B domain or with control lentivirus. Cells were harvested 72 h after infection. Total RNA from the cells was used to quantify mRNA levels for the indicated genes that were known to be either ELK1-dependent or ARE-dependent for activation by AR. The *inset* shows cell lysates probed by Western blotting using an antibody to the amino-terminal domain of AR or with antibody to GAPDH (loading control). *G,* hormone-depleted LNCaP cells transduced using lentivirus expressing either the AR A/B domain or with control lentivirus. After 72 h, cells were plated in 96-well plates, and cell growth was monitored by the MTT assay. The vector control cells were treated with R1881 (1 nm) or vehicle 24 h after plating. The *inset* shows Western blotting analysis of cell lysates, 72 h post-infection, using antibody to the amino-terminal domain of AR or with antibody to GAPDH (loading control). For all transfections, a *Renilla* luciferase reporter was used as the control for transfection efficiency. In all panels, the *error bars* represent standard deviation of experimental triplicates. *, *p* < 0.001.

AR includes the following: (i) an amino-terminal A/B domain containing ligand-independent transcriptional activation functions (AF1 and AF5); (ii) a carboxyl-terminal region (E and F domains) containing the ligand binding pocket and a ligand-dependent activation function (AF2); and (iii) internal DNA binding and hinge domains (C and D domains) (Fig. 1*A*). Typically (*e.g.* in LNCaP cells), the binding of androgen is necessary for AR to localize in the nucleus, to associate with classical androgen-response elements (AREs), and to activate its gene targets. However, AR has splice variants with carboxyl-terminal truncations that remove the ligand binding pocket; such variants are known to activate growth genes and support growth of PCa cells in a hormone-independent manner. Therefore, we tested the ability of the A/B domain of AR to induce ELK1-dependent gene activation. In contrast to full-length AR, the AR A/B domain does not require bound hormone for nuclear localization.

We first used a minimal TATA-dependent promoter luciferase construct in which two ELK1-binding cis-elements were placed upstream of the TATA box ((*ELK1*)_2_-*TATA-LUC*). This construct was transfected along with an expression plasmid for either the full-length AR or the N-terminal A/B domain of AR into AR-negative HeLa cells. The full-length AR was able to activate the promoter in an androgen-dependent manner (Fig. 1*B*). In contrast, the A/B domain activated the promoter to a comparable extent both in the presence and in the absence of hormone (Fig. 1*B*). When the cells were transfected with the minimal promoter construct in which the ELK1-binding elements were substituted with a canonical ARE, promoter activation only occurred through the full-length AR and in the presence of androgen (Fig. 1*C*). Western blotting analysis confirmed expression of both the full-length AR and AR(A/B) in the transfected cells (Fig. 1*D*).

Next, we compared the abilities of AR(A/B) and the full-length AR to activate genes (*CDCA3*, *CDCA5, CDC6*, and *DTL*) previously shown to be activated by AR in an ELK1-dependent manner (31). We also tested for activation of genes (*PRKCA, TMPRSS2*, and *KLK3*) known to be activated by androgen (R1881) in an ARE-dependent manner. Androgen activated both classes of target genes in LNCaP cells, which express full-length AR (Fig. 1*E*). However, when AR(A/B) was ectopically expressed in LNCaP cells in the absence of androgen (Fig. 1*F*, *inset*), only the ELK1-dependent target genes were activated (Fig. 1*F*). This result demonstrates that the A/B domain of AR is able to recapitulate ELK1-dependent gene activation by androgen plus AR.

Next, we tested whether the A/B domain of AR could support hormone-independent growth in LNCaP cells. AR(A/B) was ectopically expressed in LNCaP cells by lentiviral transduction, and control cells were infected with non-expressing lentivirus (Fig. 1*G*, *inset*). Growth of the control cells was dependent on androgen (Fig. 1*G*). The A/B domain of AR was capable of supporting androgen-independent cell growth, albeit less robustly than androgen (Fig. 1*G*).

Collectively, the data in Fig. 1 demonstrate that the amino-terminal A/B domain of AR is adequate for cooperation with ELK1 and for ELK1-dependent transcriptional activation. This hormone-independent action of the A/B domain is associated with partial recapitulation of androgen-dependent growth induced by full-length AR. The A/B domain may represent the minimal structural unit of AR that is required for synergizing with ELK1.

##### Mapping the Region(s) in ELK1 Required for Association with AR(A/B)

ELK1 consists of several functional domains. The amino terminus comprises the A domain encompassing the ETS DNA binding domain (45). The B domain interacts with SRF and directs ternary complex formation (46). The C domain resides at the carboxyl terminus of the protein and regulates activation through phosphorylation by mitogen-activated protein kinases (MAPKs). The D domain is a docking site for MAPKs. There is also an additional MAPK-docking site within the C domain, known as the DEF (docking site for ERK, F*X*FP) motif (47–49). Segments of ELK1 that are required for association with the AR A/B domain were mapped using a mammalian two-hybrid assay. For the two-hybrid assay, we used HeLa cells in which a minimal promoter-luciferase reporter construct with Gal4 elements upstream of the TATA box (*GAL4-TATA-LUC*) was stably integrated. These cells also stably expressed the AR(A/B)-VP16 fusion protein. The recombinant HeLa cells were transfected with Gal4-ELK1 fusion constructs in which the DNA binding domain (amino acids 1–86) was replaced by that of Gal4. The reporter readout was used to assess the ability of the Gal4-ELK1 fusion proteins to associate with AR(A/B)-VP16. In parallel, HeLa cells stably expressing *GAL4-TATA-LUC* alone were co-transfected with each one of the Gal4-ELK1 constructs and an expression plasmid for a constitutively active mutant of MEK1 (CA-MEK1). Activation of *GAL4-TATA-LUC* by CA-MEK1 entails phosphorylation and functional association of ERK1/2 with Gal4-ELK1; therefore, this parallel test probes the ability of each Gal4-ELK1 deletion/mutation construct to associate with and become activated by ERK1/2.

**FIGURE 2.**
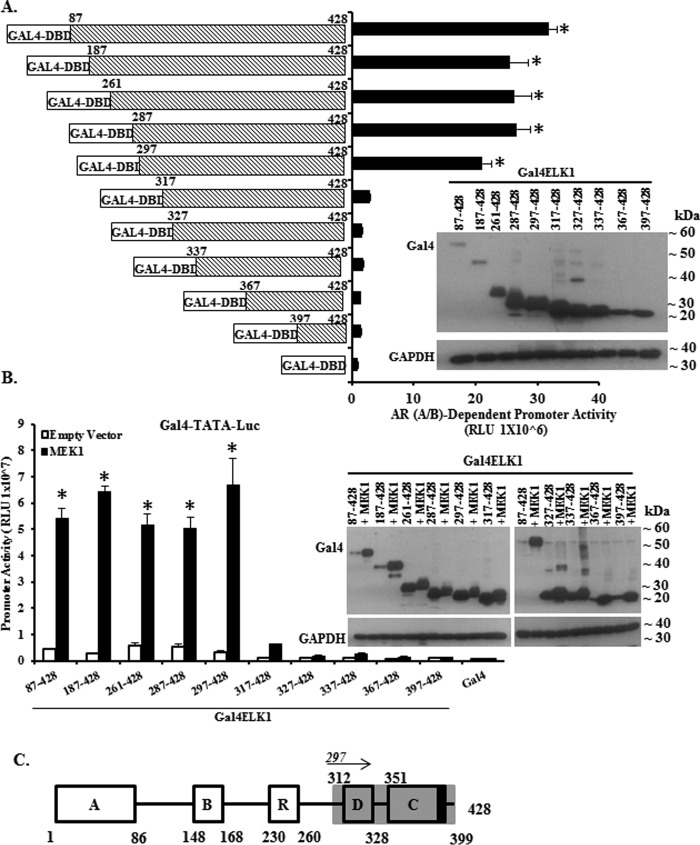
**Mapping ELK1 polypeptide segments required for co-activation by AR(A/B) by amino-terminal deletion analysis.** *A* shows data obtained using recombinant HeLa cells generated by stably transducing a minimal promoter-luciferase reporter containing upstream Gal4 elements (*GAL4-TATA-LUC*) and also with a vector expressing the AR A/B domain fused to the VP16 transactivation domain. Cells were transfected with plasmids expressing Gal4 fusion proteins of ELK1. The fusion constructs substituted the Gal4 DNA binding domain (Gal4-DBD) for the ETS DNA binding domain of ELK1. Within this fusion construct, a series of amino-terminal deletions were made, as indicated in the schematic in *A*. Forty eight hours after transfection with the various Gal4-ELK1fusion constructs, cells were harvested by preparing lysates for measurement of luciferase activity. The promoter activity shown on the *y* axis required the presence of the AR A/B domain as knocking down AR(A/B) expression in the same cells transfected with full-length Gal4-ELK1 decreased the promoter activity to the basal value shown in the figure for Gal4-DBD alone. The *inset* shows cell lysates probed by Western blotting with antibodies against Gal4 or GAPDH (loading control). *B* shows data obtained using recombinant HeLa cells generated by stably transducing only *GAL4-TATA-LUC*. The cells were transfected with each of the Gal4-ELK1 fusion constructs used in *A* and co-transfected with an expression plasmid for a constitutively active mutant of MEK1 or with the vector control. Forty eight hours after transfection with the various Gal4-ELK1 fusion constructs, cells were harvested by preparing lysates for measurement of luciferase activity. The *inset* shows cell lysates probed by Western blotting with antibodies against Gal4 or GAPDH (loading control). *C* shows a schematic of the domain organization of ELK1; here, the amino-terminal deletion mapping of an ELK1 polypeptide segment encompassing residues required for association with AR(A/B) (data from *A*) is represented by *gray shading*. For all transfections, a *Renilla* luciferase reporter was used as the control for transfection efficiency. In all panels, the *error bars* represent standard deviation of experimental triplicates. *, *p* < 0.001.

First, Gal4-ELK1 constructs containing progressive deletions beginning from the amino terminus were tested in the two-hybrid assay (Fig. 2*A*). Expression of the transfected constructs was confirmed by Western blotting, using antibody to the Gal4 DNA binding domain (Fig. 2*A*, *inset*). The reporter assay values were similar to the full-length construct for deletions to positions 187, 261, 287, and 297. However, there was virtually a complete loss of reporter activity for deletions to positions 317, 327, 337, 367, and 397. These results map an element required for association of AR(A/B) to the region downstream of amino acid residue 297 of ELK1 (Fig. 2, *A* and *schematic* in *C*). The parallel experiment testing activation of the Gal4-ELK1 constructs by CA-MEK1, showed a virtually identical pattern of activity (Fig. 2*B, inset*), a result that is consistent with the position of the D-box region beginning at residue 312 (*schematic* in Fig. 2*C*).

**FIGURE 3.**
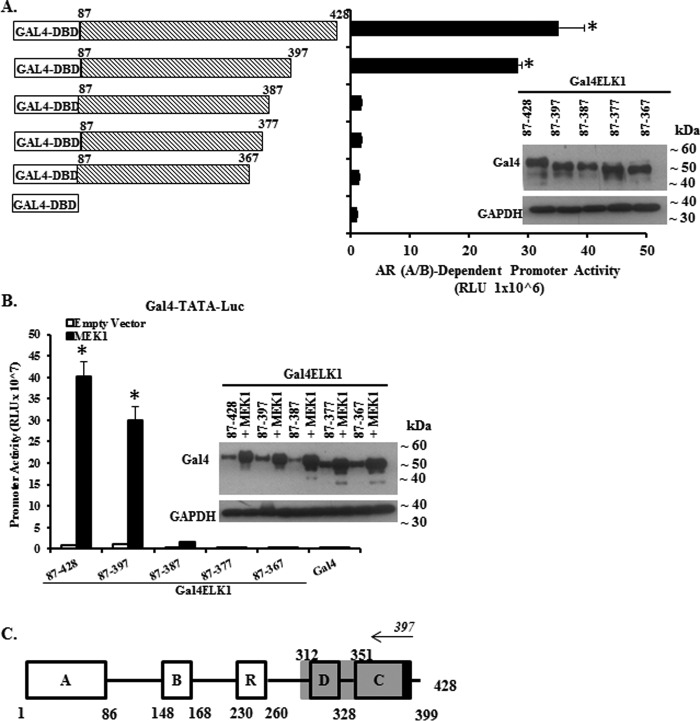
**Mapping ELK1 polypeptide segments required for co-activation by AR(A/B) by carboxyl-terminal deletion analysis.** *A* shows data obtained using recombinant HeLa cells generated by stably transducing a minimal promoter-luciferase reporter containing upstream Gal4 elements (*GAL4-TATA-LUC*) and also with a vector expressing the AR A/B domain fused to the VP16 transactivation domain. Cells were transfected with plasmids expressing Gal4 fusion proteins of ELK1. The fusion constructs substituted the Gal4 DNA binding domain (*Gal4-DBD*) for the ETS DNA binding domain of ELK1. Within this fusion construct, a series of carboxyl-terminal deletions were made, as indicated in the schematic in *A*. Forty eight hours after transfection with the various Gal4-ELK1 fusion constructs, cells were harvested by preparing lysates for measurement of luciferase activity. The promoter activity shown on the *y* axis required the presence of the AR A/B domain because knocking down AR(A/B) expression in the same cells transfected with full-length Gal4-ELK1 decreased the promoter activity to the basal value shown in the figure for Gal4-DBD alone. The *inset* shows cell lysates probed by Western blotting with antibodies against Gal4 or GAPDH (loading control). *B* shows data obtained using recombinant HeLa cells generated by stably transducing only *GAL4-TATA-LUC*. The cells were transfected with each of the Gal4-ELK1 fusion constructs used in *A* and co-transfected with an expression plasmid for a constitutively active mutant of MEK1 or with the vector control. Forty eight hours after transfection with the various Gal4-ELK1 fusion constructs, cells were harvested by preparing lysates for measurement of luciferase activity. The *inset* shows cell lysates probed by Western blotting with antibodies against Gal4 or GAPDH (loading control). *C* shows a schematic of the domain organization of ELK1; here, the carboxyl-terminal deletion mapping of an ELK1 polypeptide segment encompassing residues required for association with AR(A/B) (data from *A*) is represented by *gray shading*. For all transfections, a *Renilla* luciferase reporter was used as the control for transfection efficiency. In all panels, the *error bars* represent standard deviation of experimental triplicates. *, *p* < 0.001.

Next, Gal4-ELK1 constructs containing progressive deletions beginning from the carboxyl terminus were tested in the two-hybrid assay (Fig. 3*A*). Again, expression of the transfected constructs was confirmed by Western blotting (Fig. 3*A*, *inset*). The reporter assay values were similar to the full-length construct for the deletion made at position 397, but it was lost for deletions to positions 387, 377, and 367. These results map an element required for association of AR(A/B) to the region upstream of amino acid residue 397 of ELK1 (Fig. 3, *A* and *schematic* in *C*). The parallel experiment testing activation of the Gal4-ELK1 constructs by CA-MEK1 again showed the same pattern (Fig. 3*B, inset*). This result is consistent with the position of the DEF motif, which serves as an additional ERK-docking site (*schematic* in Fig. 3*C*).

**FIGURE 4.**
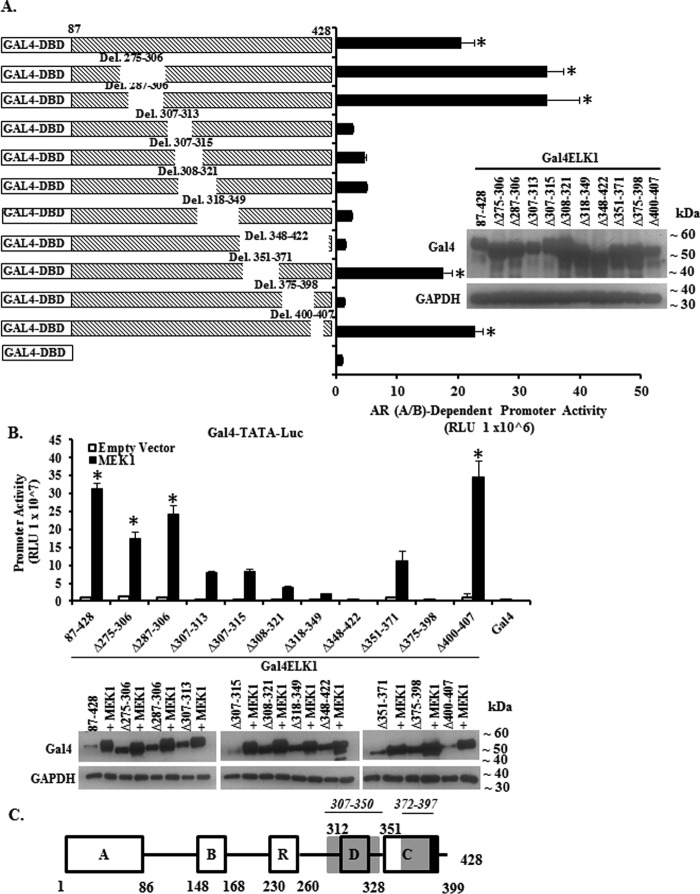
**Mapping ELK1 polypeptide segments required for co-activation by AR(A/B) by internal deletion analysis.** *A* shows data obtained using recombinant HeLa cells generated by stably transducing a minimal promoter-luciferase reporter containing upstream Gal4 elements (*GAL4-TATA-LUC*) and also with a vector expressing the AR A/B domain fused to the VP16 transactivation domain. Cells were transfected with plasmids expressing Gal4 fusion proteins of ELK1. The fusion constructs substituted the Gal4 DNA binding domain (*Gal4-DBD*) for the ETS DNA binding domain of ELK1. Within this fusion construct, a series of internal deletions were made, as indicated in the schematic in *A*. Forty eight hours after transfection with the various Gal4-ELK1 fusion constructs, cells were harvested by preparing lysates for measurement of luciferase activity. The promoter activity shown on the *y* axis required the presence of the AR A/B domain because knocking down AR(A/B) expression in the same cells transfected with full-length Gal4-ELK1 decreased the promoter activity to the basal value shown in the figure for Gal4-DBD alone. The *inset* shows cell lysates probed by Western blotting with antibodies against Gal4 or GAPDH (loading control). *B* shows data obtained using recombinant HeLa cells generated by stably transducing only *GAL4-TATA-LUC*. The cells were transfected with each of the Gal4-ELK1 fusion constructs used in *A* and co-transfected with an expression plasmid for a constitutively active mutant of MEK1 or with the vector control. Forty eight hours after transfection with the various Gal4-ELK1 fusion constructs, cells were harvested by preparing lysates for measurement of luciferase activity. The *inset* shows cell lysates probed by Western blotting with antibodies against Gal4 or GAPDH (loading control). *C* shows a schematic of the domain organization of ELK1; here, the deletion mapping of two ELK1 polypeptide segments encompassing residues required for association with AR(A/B) (data from Figs. 3*A*, 4*A,* and 5*A*) is represented by *gray shading* of the two segments. For all transfections, a *Renilla* luciferase reporter was used as the control for transfection efficiency. In all panels, the *error bars* represent standard deviation of experimental triplicates. *, *p* < 0.001.

Additionally, we made and tested the effects of a series of short overlapping internal deletions within Gal4-ELK1 (Fig. 4*A*) using the two-hybrid assay. The internal deletions covered segments flanking and within the region mapped above by amino- and carboxyl-terminal deletion analyses. Expression of the Gal4-ELK1 constructs was confirmed by Western blotting (Fig. 4*A*, *inset*). As shown in Fig. 4*A*, the reporter assay values for the constructs clearly fell into the following two groups: those corresponding to the activity of the full-length construct, and those approaching the background value obtained by transfecting the Gal4 DNA binding domain alone. Based on these data, we were able to confirm and further bracket peptide motifs required for association of AR(A/B) to within the amino acid sequences 307–350 and 372–397. Activation of the Gal4-ELK1 internal deletion constructs by CA-MEK1 followed a similar pattern except for the deletion 351–371, which reduced activation by CA-MEK1 but not association with AR(A/B)-VP16 (Fig. 4*B, inset*). These results are again consistent with the locations of the ERK-docking sites in ELK1 (D box and DEF motif) (*schematic* in Fig. 4*C*). As residues 351–371 lie within the transactivation domain (domain C, residues 351–399) of ELK1 (*schematic* in Fig. 4*C*) and include two ERK phosphorylation sites (39), its requirement for optimal activation of ELK1 by CA-MEK1 was anticipated.

**FIGURE 5.**
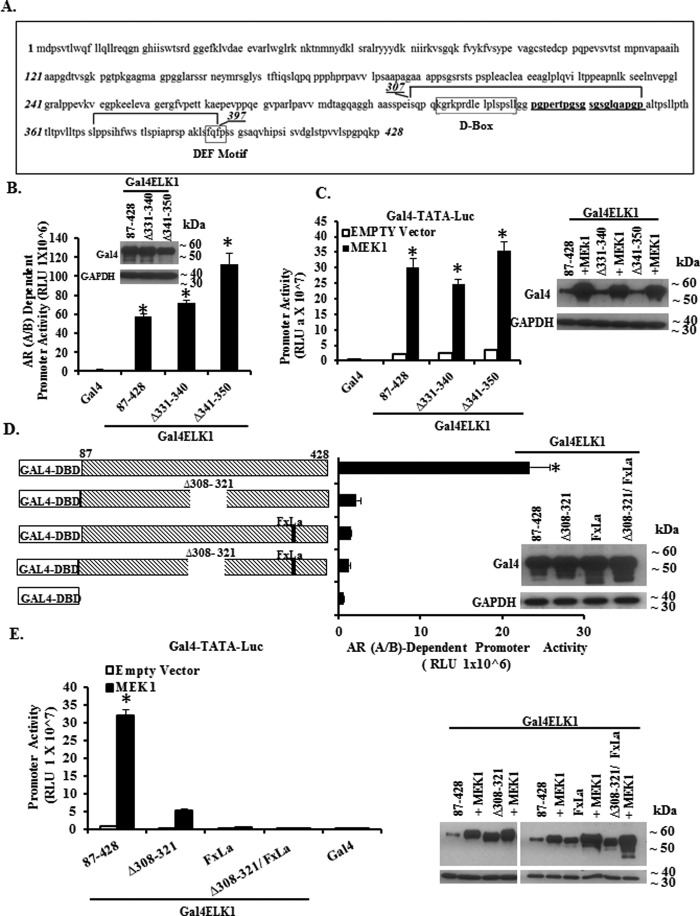
**Further refinement of the mapping of ELK1 motifs required for co-activation by AR(A/B).** *A* shows the ELK1 polypeptide sequence. The *bracketed* segments indicate the two segments that were mapped from the deletion analyses in Figs. 3–5 as regions containing residues essential for the association of ELK1 with AR(A/B). The *boxed* segments denote the D-domain of ELK1 and the F*X*FP motif of ELK1. The segments in *bold font* represent peptides that were deleted for further mapping in *B* and *C. B* and *D* show data obtained using recombinant HeLa cells generated by stably transducing a minimal promoter-luciferase reporter containing upstream Gal4 elements (*GAL4-TATA-LUC*) and also with a vector expressing the AR A/B domain fused to the VP16 transactivation domain. Cells were transfected with plasmids expressing Gal4 fusion proteins of ELK1. The fusion constructs substituted the Gal4 DNA binding domain (*Gal4-DBD*) for the ETS DNA binding domain of ELK1. Within this fusion construct, the indicated internal deletions or mutations were made, as indicated in the schematics in *B* and *D*. Forty eight hours after transfection with the various Gal4-ELK1 fusion constructs, cells were harvested by preparing lysates for measurement of luciferase activity. The promoter activity shown on the *y* axis required the presence of the AR A/B domain as knocking down AR(A/B) expression in the same cells transfected with full-length Gal4-ELK1 decreased the promoter activity to the basal value shown in the figure for Gal4-DBD alone. The *insets* in *B* and *D* show cell lysates probed by Western blotting with antibodies against Gal4 or GAPDH (loading control). *C* and *E* show data obtained using recombinant HeLa cells generated by stably transducing only *GAL4-TATA-LUC*. The cells were transfected with each of the Gal4-ELK1 fusion constructs used in *B* and *D*, respectively, and co-transfected with an expression plasmid for a constitutively active mutant of MEK1 or with the vector control. Forty eight hours after transfection with the various Gal4-ELK1fusion constructs, cells were harvested by preparing lysates for measurement of luciferase activity. The *insets* in *C* and *E* show cell lysates probed by Western blotting with antibodies against Gal4 or GAPDH (loading control). For all transfections, a *Renilla* luciferase reporter was used as the control for transfection efficiency. In all panels, the *error bars* represent standard deviation of experimental triplicates. *, *p* < 0.001.

To further refine the mapping data for the motif required for association of AR(A/B) within the ELK1 polypeptide segment 307–350, we made additional deletions of residues 331–340 and 341–350 (Fig. 5*A*). Western blotting confirmed expression of the constructs (Fig. 5*B*, *inset*). Neither deletion reduced the reporter activity in the two-hybrid assay (Fig. 5*B*), thus further bracketing the upstream element required for association of AR(A/B) to within amino acid residues 307–330. These internal deletions also had no effect on activation of Gal4-ELK1 by CA-MEK1 (Fig. 5*C, inset*). Finally, we tested whether the FQFP motif (F*X*FP motif) and the D-box region (residues 308–321), both of which direct ERK docking to ELK1, were also required for the association of AR(A/B) to ELK1. We tested a Gal4-ELK1 construct in which the FQFP motif was mutated to FQLA, a Gal4-ELK1 construct in which the D-box region (ELK1 residues 308–321) was deleted, and a Gal4-ELK1 construct with both lesions. All three resulted in loss of function of AR(A/B)-VP16 in the two-hybrid assay (Fig. 5*D, inset*), mirroring the loss of activation by CA-MEK1 (Fig. 5*E, left* and *right*).

Collectively, the data from Figs. 2 to 5 identify two peptide motifs in ELK1 that are both essential for its association with the A/B domain of AR. The pattern of retention or loss of association with AR(A/B) due to the various deletions/mutations in ELK1 mirrored the pattern for activation by ERK with one exception, *i.e.* a deletion within the transactivation domain of ELK1, which disrupted activation by ERK but did not affect association with AR(A/B). The two ELK1 motifs required for association with AR(A/B) equate to the two ERK-docking sites in ELK1.

##### ELK1 Motifs Required for Association with the AR A/B Domain Participate in Hormone-induced Activation of ELK1 by Full-length AR

**FIGURE 6.**
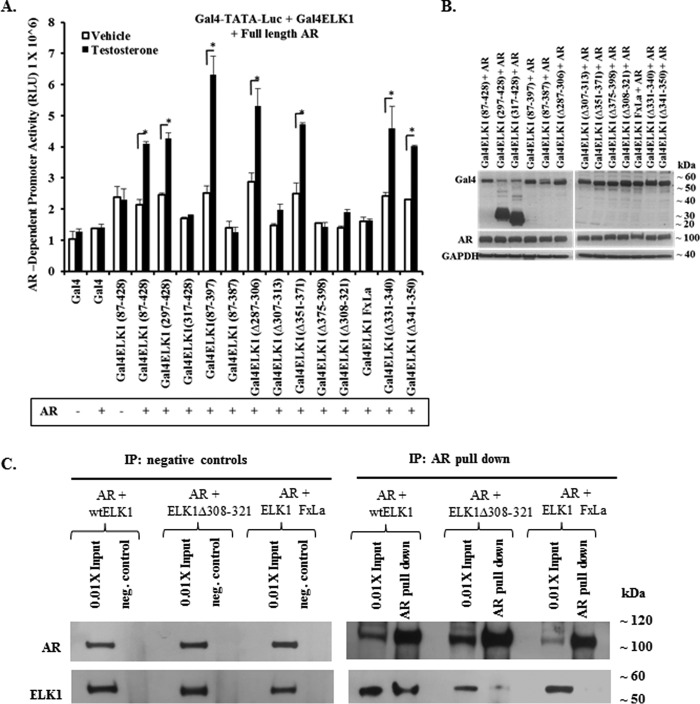
**ELK1 motifs required for co-activation by full-length AR.** *A* shows data obtained using recombinant HeLa cells generated by stably transducing a minimal promoter-luciferase reporter containing upstream Gal4 elements (*GAL4-TATA-LUC*). Cells were plated in hormone-depleted media and co-transfected with expression plasmids for the indicated Gal4-ELK1 fusion proteins and an expression plasmid for full-length AR. The fusion constructs substituted the Gal4 DNA binding domain (Gal4-DBD) for the ETS DNA binding domain of ELK1. At the time of transfection, the cells were treated with testosterone (10 nm) or vehicle control. Forty eight hours after transfection, cells were harvested by preparing lysates for measurement of luciferase activity. *B* shows cell lysates probed by Western blotting with antibodies against Gal4, AR, or GAPDH (loading control). *C* shows data on co-immunoprecipitation of ectopic AR and ectopic ELK1 or ELK1 mutants from HeLa cell lysates. HeLa cells were transfected with the expression plasmid for AR and co-transfected with an expression plasmid for WTELK1 or one of two ELK1 mutants, ELK1Δ308–321 and ELK1 FxLa. The lysates were immunoprecipitated (*IP*) using either antibody to AR or a negative control. The immunoprecipitates were probed by Western blotting using antibody to either AR or ELK1 as indicated. *, *p* < 0.01.

To further validate the mapping of ELK1 motifs required for association with AR, it was necessary to confirm that the mapping data obtained above by the mammalian two-hybrid assay using AR(A/B)-VP16 applies to the full-length AR. For this purpose we tested Gal4-ELK1 fusion constructs with deletions and mutations that were indicative for bracketing the binding sites for AR(A/B) (Fig. 6*A*). Each of these constructs, as well as control constructs, was co-transfected with full-length AR in recombinant HeLa cells in which the *GAL4-TATA-LUC* promoter-reporter was stably integrated. The cells were treated with testosterone or the vehicle control beginning at the time of transfection. Androgen activated the promoter only in cells transfected with Gal4-ELK1 constructs that were found in the preceding sections to have the ability to bind AR(A/B) (Fig. 6*A*). It was confirmed that both AR and the appropriate Gal4-ELK1 construct were expressed in the transfected cells, as observed by Western blotting using antibody to either Gal4 or AR (Fig. 6*B*).

To complement the above data, we co-expressed in HeLa cells AR and WTELK1 or AR and a mutant of ELK1 in which either one of the two ERK-docking sites was disrupted. AR and WTELK1 co-immunoprecipitated in these cells; however, when ELK1 was mutated at either one of its two ERK-docking sites, it was unable to co-immunoprecipitate with AR (Fig. 6*C*). These results demonstrate that functional association of the full-length AR with ELK1 (or transcriptional co-activation of ELK1 by AR) requires the same ELK1 motifs as those mapped for the binding of AR(A/B) to ELK1.

##### Influence of SRF on the Interaction of AR with ELK1

**FIGURE 7.**
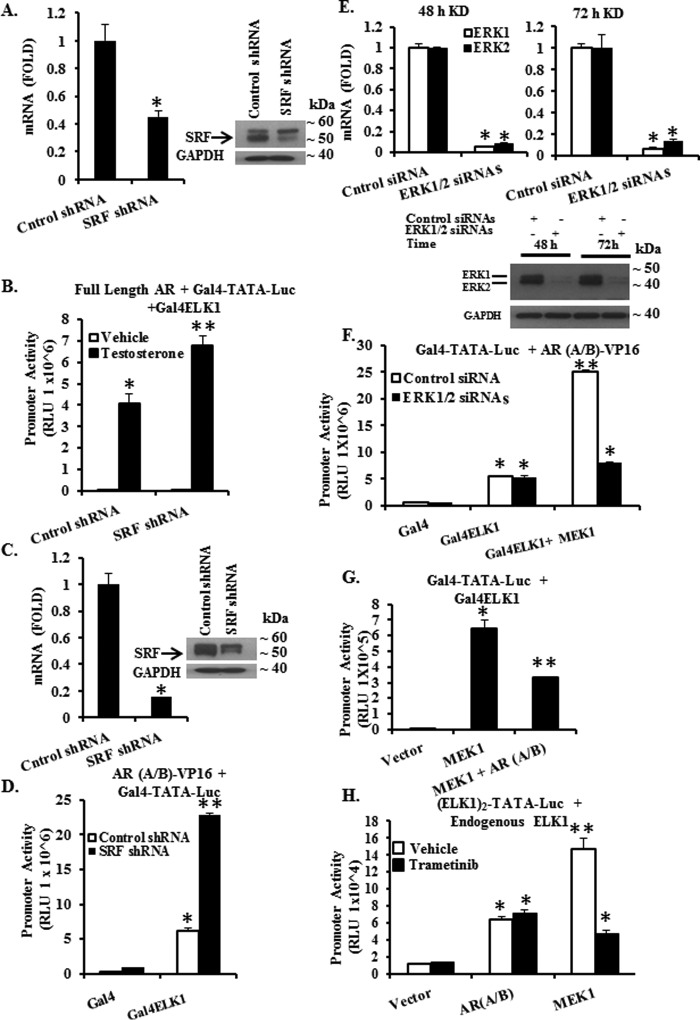
**Effect of depleting SRF or ERK1/2 on the interactions of AR with ELK1.** *A* and *B* show data obtained using recombinant HeLa cells generated by stably transducing a minimal promoter-luciferase reporter containing upstream Gal4 elements (*GAL4-TATA-LUC*) and also with vectors expressing AR and a Gal4-ELK1 fusion construct in which the Gal4 DNA binding domain was substituted for the ETS DNA binding domain of ELK1. The cells were depleted of hormone and then transduced with shRNA against SRF or a non-targeted control shRNA using lentivirus. Seventy two hours after infection, cells were treated with testosterone (10 nm) or vehicle for a further 48 h. The cells were then harvested to quantify SRF mRNA (*A*) or for Western blotting analysis using antibody to SRF or to GAPDH (loading control) (*A, inset*) or for luciferase activity (*B*). *C* and *D* show data obtained using recombinant HeLa cells generated by stably transducing *GAL4-TATA-LUC* and also a vector expressing the AR A/B domain fused to the VP16 transactivation domain (AR(A/B)-VP16). The cells were transduced with shRNA against SRF or a non-targeted control shRNA using lentivirus. Seventy two hours after infection, the cells were transfected with expression plasmid for the Gal4-ELK1 fusion protein. Forty eight hours later, the cells were harvested to quantify SRF mRNA (*C*) or for Western blotting analysis using antibody to SRF or to GAPDH (loading control) (*C, inset*) or to measure luciferase activity (*D*). *E–G* show data obtained using the recombinant HeLa cells with stably incorporated *GAL4-TATA-LUC* and stably expressing the AR(A/B)-VP16 fusion protein. Cells were transfected with a mixture of siRNA against ERK1 and ERK2 or with control non-targeted siRNA. After 48 h of transfection, the cells were harvested to quantify mRNAs for ERK1 and ERK2 (*E*) or for Western blotting analysis using antibody to ERK1/2 or to GAPDH (loading control) (*E, below*); the remaining cells were transfected for a second time with the Gal-4ELK1 expression plasmid or the control vector plasmid or the plasmid for constitutively active mutant of MEK1 (*F*). After a further 24 h, the cells were harvested to quantify mRNAs for ERK1 and ERK2 (*E*) or for Western blotting analysis using antibody to ERK1/2 or to GAPDH (loading control) (*E, below*) or to measure luciferase activity (*F*). *H,* HeLa cells were co-transfected with an ELK1-driven minimal promoter-luciferase reporter ((*ELK1*)_2_-*TATA-LUC*) and expression plasmid for either AR(A/B) or constitutively active MEK1 or control vector plasmid. The cells were treated with trametinib (1 μm) or vehicle for 48 h beginning with the time of transfection. Luciferase activity was measured in the cell lysates. For all transfections, a *Renilla* luciferase reporter was used as the control for transfection efficiency. In all panels, the *error bars* represent standard deviation of experimental triplicates. *, *p* < 0.001; **, *p* < 0.001.

Functional interactions between AR and ELK1 could be influenced by the association of ELK1 with its DNA-binding partner SRF. To explore this possibility, we first used recombinant HeLa cells in which a minimal promoter-luciferase reporter construct with Gal4 elements upstream of the TATA box (*GAL4-TATA-LUC*) was stably integrated in the chromatin. The cells also stably expressed full-length AR and the Gal4-ELK1(87–428) fusion protein. Induction of reporter luciferase activity by testosterone was measured to quantify the binding of AR to ELK1. In these cells, lentiviral transduction with shRNA against SRF resulted in knocking down SRF at both the mRNA and protein levels compared with control shRNA (Fig. 7*A* and *inset*). Knockdown of SRF significantly increased testosterone-induced luciferase activity (Fig. 7*B*).

As a complementary approach, we used recombinant HeLa cells that stably expressed the AR(A/B) domain fused to the VP16 transactivation domain (AR(A/B)-VP16). Additionally, the *GAL4-TATA-LUC* promoter-reporter was stably integrated in the chromatin. In these cells, we examined the effect of SRF knockdown on the hormone-independent activation of ELK1 by AR(A/B)-VP16. Cells were transduced with shRNA against SRF or with control shRNA followed by transfection with the Gal4-ELK1 fusion construct or with the Gal4 control vector. SRF shRNA substantially decreased both SRF mRNA and protein (Fig. 7*C* and *inset*) and increased the luciferase reporter activity 3-fold compared with cells transduced with control shRNA (Fig. 7*D*). Taken together, these results demonstrate that SRF is not required for activation of ELK1 by AR; rather, SRF may hinder optimal ELK1-dependent transcriptional activation by AR.

##### Influence of ERK1/2 on the Interaction of AR with ELK1

The requirement for MAPK-docking motifs in ELK1 suggested the possibility that the functional association of AR with ELK1 could involve ERKs, the classical ELK1-activating protein kinases, as an essential component of the ELK1-AR complex. As a first test, we used recombinant HeLa cells stably expressing AR(A/B)-VP16 in which the *GAL4-TATA-LUC* promoter-reporter was also stably integrated. These cells were co-transfected with siRNAs against ERK1 and ERK2 or transfected with control siRNA. ERK mRNAs and proteins were depleted within 48 h of siRNA transfection compared with the control siRNA (Fig. 7*E*, *top* and *bottom*). Following knockdown of ERKs, the cells were transfected again, this time with expression plasmid for the Gal4-ELK1 fusion protein or the control Gal4 vector. In parallel, cells were also co-transfected with Gal4-ELK1 and the expression plasmid for CA-MEK1. The reporter luciferase activity was measured 24 h following the second transfection (72 h following the first transfection), while the knockdown of ERKs persisted (Fig. 7*E*, *top* and *bottom*). The synergistic activation of the promoter-reporter by AR and ELK1 was unaffected by the combined depletion of ERK1 and ERK2 (Fig. 7*F*). In contrast, the CA-MEK-induced hyper-activation of the promoter was abrogated by depletion of ERK1 and ERK2, decreasing the promoter activation to the level observed for cells only expressing AR(A/B) and Gal4-ELK1 (Fig. 7*F*).

Second, we tested whether AR(A/B) would compete with ERKs as activators of ELK1. We used recombinant HeLa cells in which the *GAL4-TATA-LUC* promoter-reporter was stably integrated and that also stably expressed the Gal4-ELK1 fusion protein. As expected, in these cells ectopic expression of CA-MEK1 strongly stimulated expression of the luciferase reporter (Fig. 7*G*). However, co-expression of AR(A/B) decreased the promoter activation (Fig. 7*G*), indicating that AR interferes with hyper-activation of ELK1 by ERKs.

In a third approach, the effect of ERK activity on the interaction between ELK1 and AR was tested using HeLa cells co-transfected with the (ELK1)_2_-TATA-luc reporter and an expression plasmid for either AR(A/B) or CA-MEK1. The (ELK1)_2_-TATA-luc promoter was responsive to the endogenous ELK1 in the cells. Following the transfections, the cells were treated with the MEK inhibitor, trametinib. As expected, MEK-induced activation of the promoter was inhibited by trametinib (Fig. 7*H*). In contrast, trametinib did not affect activation of the promoter by AR(A/B). The results from the complementary approaches of depletion and inhibition of ERK1/2 and competition between MEK1 and AR(A/B) as activators of ELK1 all demonstrate that the functional interaction of AR and ELK1 is insensitive to the expression or activation status of ERKs. The data indicate that the association of AR with ELK1 occurs independently of ERK1 and ERK2.

##### Direct Binding of AR and ELK1

**FIGURE 8.**
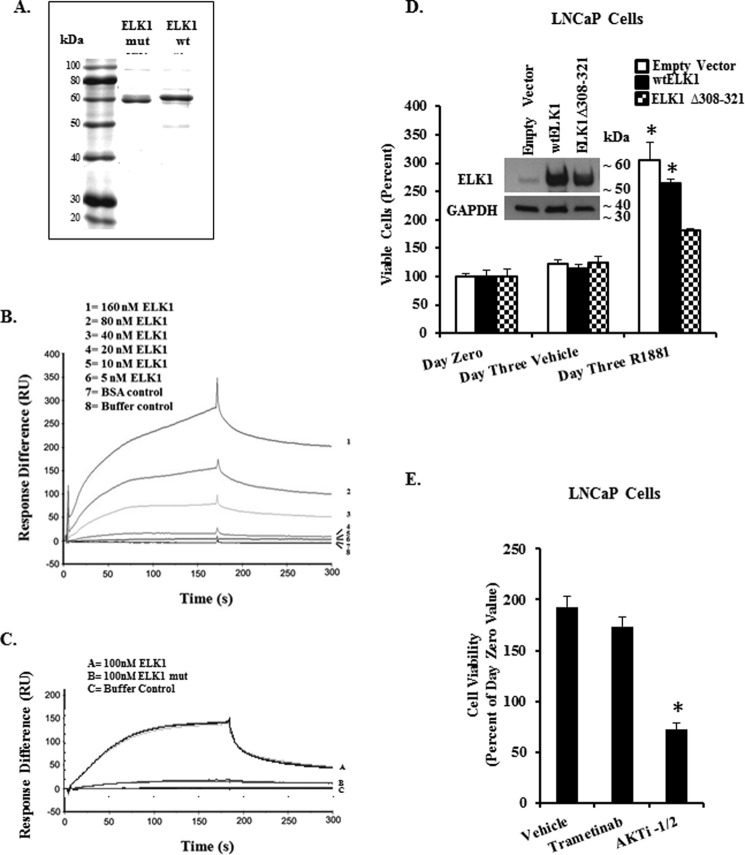
**Direct binding of ELK1 and AR and the effect of disrupting docking sites in ELK1 on AR binding and androgen-dependent cell growth.** *A* shows SDS-PAGE of purified His-tagged ELK1 protein and His-tagged ELK1 protein mutated (*ELK1 mut*) in both D-box (Δ308–321) and DEF motif. The protein bands were visualized by Coomassie Blue staining and estimated to be >85% pure and used in the SPR experiments below together with purified AR obtained commercially. *B* shows SPR kinetic curves for quantitative analyses of AR binding to His-tagged ELK1. AR was immobilized on a CM5 sensor chip, and ELK1 was diluted in a series of concentrations (0, 5, 10, 20, 40, 80, and 160 nm). The results were normalized by subtracting the SPR response (*RU*) for buffer alone or BSA and performed in duplicate. *C* shows SPR kinetic curves for quantitative analyses of AR binding to His-tagged mutant ELK1. AR was immobilized as in *B,* and ELK1 or mutant ELK1 was used at 100 nm. The kinetic curves for triplicate determinations are shown. *D,* hormone-depleted LNCaP cells were transduced using lentivirus expressing either the WTELK1, or ELK1(Δ308–321), or with control lentivirus. After 72 h, cells were plated in 96-well plates, and cell growth was monitored by the MTT assay. Twenty four hours after plating, the cells were treated with R1881 (1 nm) or vehicle for a further 72 h. The *inset* in *D* shows Western blotting analysis of cell lysates, 72 h post-infection, using antibody to ELK1 or with antibody to GAPDH (loading control). The *error bars* represent standard deviation of experimental triplicates. *, *p* < 0.001. *E,* hormone-depleted LNCaP cells were plated in 96-well plates in the presence of R1881 (1 nm) together with vehicle, trametinib (1 μm), or AKTi-1/2 (2 μm). MTT assay was performed 72 h later. The *error bars* represent standard deviation of experimental triplicates. *, *p* < 0.001.

The studies above indicate that the classical binding partners of ELK1, *i.e.* SRF and ERK1/2, interfere with rather than facilitate functional association of AR with ELK1. Therefore, to test whether the association of AR with ELK1 could be due to direct binding, we used surface plasmon resonance (SPR). Purified AR (Abcam, Cambridge, MA) was immobilized as the “ligand.” His-tagged ELK1 and His-tagged mutant ELK1, in which both the D-box and the DEF motif were disrupted (Δ308–321, F397L, and P398A), were affinity-purified to >85% (Fig. 8*A*) and used as the analyte. The binding kinetics for ELK1 was determined at concentrations of 0, 5, 10, 20, 40, 80, and 160 nm (Fig. 8*B*). Bovine serum albumin was used as the negative control. Kinetic constants were evaluated using the BIAevaluation software. The equilibrium dissociation constant for the AR-ELK1 interaction was determined to be 1.9 × 10^−8^ m. When the mutant ELK1 was used as the analyte at a concentration of 100 nm, the average response units from triplicate measurements was reduced by ∼90% compared with 100 nm ELK1 (Fig. 8*C*). The relatively high affinity of binding of purified preparations of AR and ELK1 strongly supports the concept that the *in situ* interactions of the two proteins are due to direct binding. Moreover, loss of this binding due to disruption of the ERK-docking sites in ELK1 strongly indicates that these are also the docking sites for AR.

##### Relevance of the Physical Association of ELK1 and AR to Androgen-dependent Cell Growth

Previous studies using ELK1 depletion methods have demonstrated that both prostate and bladder cancer cells require ELK1 for androgen-dependent growth (31–33). However, they did not test the physiological effect of disrupting the association of ELK1 with AR. To disrupt the hormone-dependent association of AR and ELK1 *in situ* without disrupting the ability of ELK1 to bind to DNA, we used an ELK1 mutant lacking the D-box region (amino acids 308–321) that should compete with endogenous ELK1 for binding to chromatin. We tested whether the mutant ELK1 would produce a dominant-negative effect on androgen-dependent growth. LNCaP cells were transduced with lentiviral expression vectors for either ELK1 or the ELK1Δ308–321 mutant (Fig. 8*D*, *inset*). Ectopic overexpression of ELK1 did not appreciably influence androgen (R1881)-dependent growth compared with the vector-transduced control (Fig. 8*D*). In contrast, ectopic overexpression of the ELK1Δ308–321 mutant showed a dominant-negative effect by inhibiting hormone-dependent growth. To confirm that the dominant-negative effect of this ELK1Δ308–321 on androgen-dependent growth was not due to dependence on activation of ELK1 by ERK, we demonstrate that LNCaP growth is insensitive to inhibition of MEK by trametinib (Fig. 8*E*); as a control in Fig. 8*E*, the AKT inhibitor AKTi-1/2 completely inhibited androgen-dependent growth. These results further validate the model that docking of AR on ELK1 is an essential component of growth signaling by androgen/AR in prostate cancer cells.

##### Synergy between the Splice Variant AR-V7 and ELK1 and AR-V7-dependent Cell Growth

**FIGURE 9.**
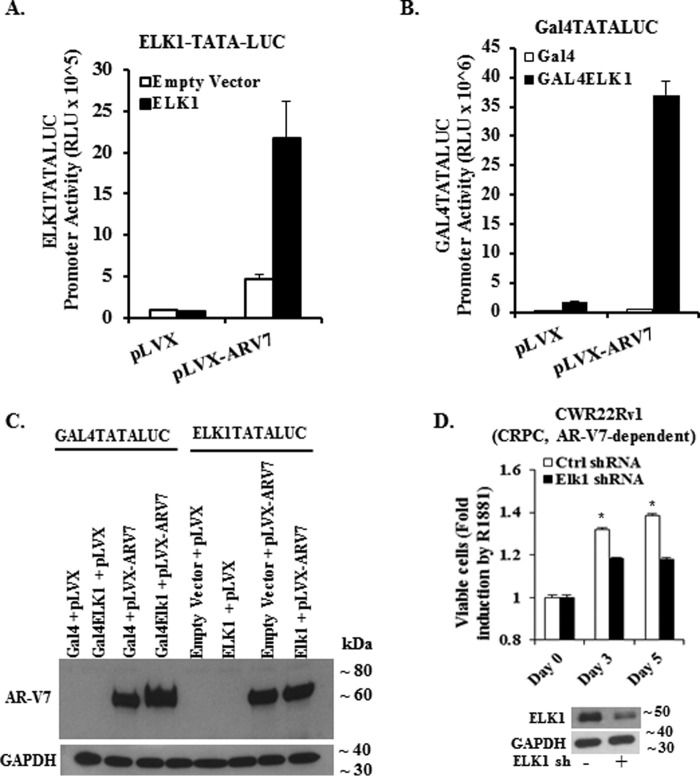
**Functional association of ELK1 and AR-V7 and effect on cell growth.** *A,* HeLa cells were co-transfected with an ELK1-driven minimal promoter-luciferase reporter ((*ELK1*)_2_-*TATA-LUC*) and expression plasmid for either AR-V7 or WTELK1 or control vector plasmid for 48 h. Luciferase activity was measured in the cell lysates. For all transfections, a *Renilla* luciferase reporter was used as the control for transfection efficiency. *B,* HeLa cells were co-transfected with a Gal4-driven minimal promoter-luciferase reporter (Gal4-TATA-Luc) and expression plasmid for either AR-V7 or Gal4-ELK1 or control vector plasmid for 48 h. Luciferase activity was measured in the cell lysates. For all transfections, a *Renilla* luciferase reporter was used as the control for transfection efficiency. *C* shows a Western blot of HeLa cell lysates corresponding to all of the transfections in *A* and *B,* which was probed using an antibody to the amino-terminal domain of AR or with antibody to GAPDH (loading control). *D, top panel* shows the effect of depleting ELK1 by lentiviral shRNA transduction on the growth of CWR22Rv1 cells monitored by the MTT assay compared with control shRNA. The Western blot in the *bottom panel* shows ELK1 shRNA-induced depletion of ELK1 compared with control shRNA; GAPDH was probed as the loading control.

The ability of the amino-terminal A/B domain of AR to synergize with ELK1 suggested that AR-V7, the major splice variant of AR, would most likely also synergize with ELK1. To test this possibility, we co-transfected the (ELK1)_2_-TATA-luc promoter-reporter construct with an expression plasmid for AR-V7 and WTELK1 into the AR-negative HeLa cells. Ectopic AR-V7 activated the promoter-driven luciferase activity well above the basal level presumably associated with endogenous ELK1 (Fig. 9*A*). Similarly, when the *GAL4-TATA-LUC* promoter was co-transfected with Gal4-ELK1 and AR-V7, there was strong induction of promoter activity (Fig. 9*B*). Western blotting analysis confirmed expression of the AR-V7 construct in both cases (Fig. 9*C*).

We have previously demonstrated that ELK1 is necessary for androgen/AR-dependent growth of prostate cancer cells. To test whether prostate cancer cells that are dependent on AR-V7 also depend on ELK1, we used shRNA to deplete ELK1 in CWR22Rv1 cells, which depend on endogenous AR-V7. Partial depletion of ELK1 prevented the growth of these cells (Fig. 9*D*, *top* and *bottom panels*). These results confirm that the AR-V7 splice variant cooperates with ELK1 as a transcriptional co-activator in the same manner as AR(A/B) and that ELK1 is also necessary for AR-V7-dependent growth of prostate cancer cells.

## Discussion

The results of this study elucidate the nature of the interaction of the ligand-independent A/B domain of AR with ELK1 that accounts for the ELK1-dependent transcriptional activity of AR in PCa cells. Systematic mapping using a mammalian two-hybrid assay and an extensive series of ELK1 deletion and point mutants, and confirmatory co-immunoprecipitation experiments, identified the two ERK-docking motifs (D-box and DEF motif) in ELK1 as the elements essential for co-activation by AR. Nonetheless, the AR synergy with ELK1 is independent of ERKs and involves AR binding directly to ELK1. Interactions with ELK1 are required for hormone-dependent growth of PCa cells and also account for the ability of the AR A/B domain, as well as the major AR splice variant AR-V7, to support their hormone-independent growth. Indeed the results strongly support the view that synergy with ELK1, via AR docking on ELK1, is a critical component of growth signaling by AR and AR-V7.

The mammalian two-hybrid mapping data with Gal4ELK1 mutants were clear-cut. Parallel studies using a constitutively active form of MEK to activate ELK1 demonstrated that the regions required for co-activation by AR(A/B) precisely coincided with the D-box and DEF motifs in ELK1 (40–42). Activation was hormone-independent as, in contrast to full-length AR, the A/B domain is not constrained by the need for ligand binding to enter the nuclear compartment. Importantly, mapping data for the sites of interaction of the AR A/B domain with ELK1 were entirely recapitulated with full-length AR and confirmed by co-immunoprecipitation. There were two notable differences between co-activation of ELK1 by AR and activation by MEK. First, the level of ELK1 transcriptional activity induced by MEK was substantially higher than that induced by AR. This difference in magnitude is associated with the transient nature of ELK1 activation through the MAPK pathway in contrast to the constitutive ELK1-dependent activation of growth genes by AR (31). Second, an intact ELK1 transactivation domain was essential for activation by MEK but not for co-activation of ELK1 by AR. This finding indicates that AR only utilizes ELK1 for recruitment to regulatory sites in chromatin to activate growth genes and that the transcriptional activity *per se* of ELK1 is unimportant for the ELK1-AR synergy.

Complementary approaches, including one- and two-hybrid assays, promoter assays, gene knockdown, MEK inhibition, and competition assays, demonstrated that the classical binding partners of ELK1 in the ternary complex factor, *i.e.* SRF and ERKs (49), are not required for co-activation of ELK1 by AR. Indeed, SRF and ERKs both appeared to interfere with the interaction of AR with ELK1. SRF knockdown may release ELK1 from immediate early gene promoters, which are unresponsive to androgen (31). Similarly, ELK1 recruits ERKs directly to a subset of target genes (50) suggesting that ERK competes directly with AR for ELK1 binding. Indeed, SPR confirmed binding between ELK1 and AR with a relatively high affinity (*K_d_*, 1.9 × 10^−8^ m), strongly supporting the premise that the transcriptional synergy between ELK1 and AR is due to direct binding. Loss of this binding upon mutational disruption of the ERK-docking sites in ELK1 equates the ELK1 motifs required for functional association with AR with those required for direct binding of AR to ELK1.

ETS elements are commonly enriched at or in the vicinity of AR-binding sites in the chromatin (51, 52), and indeed physical association of AR has been suggested with ETV1 (53) and ETS1 (51). However, in neither case has the structural binding elements in either AR or the ETS protein been elucidated. Among the known DNA-binding proteins that have been suggested to recruit AR, the interactions of AR with HoxB13 and C/EBPa are perhaps the best studied. HoxB13 interacts with the DNA binding domain of AR (29), whereas C/EBPa did not show a strong AR domain selectivity for interaction, and its association with AR involved multiple AR domains (30). Relatively little is known about the structural basis for non-genomic interactions of AR and other nuclear receptors with signaling pathways involving MAPK, PI3K/Akt, PKC, PLC, and G-protein-coupled receptors (1). Notable exceptions include the direct or indirect associations of NRs with the Src homology 2 or 3 domains where proline-rich motifs in AR and the progesterone receptor or L*XX*L motifs in the estrogen receptor are required (54–57). The relatively large size and multidomain structure of NRs appear to offer a diversity of binding motifs for interactions with co-activators and DNA binding transcription factors.

To our knowledge, the functional interaction of AR with ELK1 is the first demonstration of a nuclear receptor co-opting protein kinase-docking sites to regulate or constitutively activate a signaling pathway. The finding that ERK-docking motifs can recruit AR suggests that AR may adopt similar modes of interaction in its cross-talk with other signaling molecules. Certainly, by mimicking ERK interactions with docking motifs, AR may interact with additional substrates of ERK, of which there are over 50 (40). There is much cross-talk between AR genomic and non-genomic pathways in prostate cancer. The canonical pathway for AR signaling requires nuclear translocation of the ligand-bound receptor to activate transcription and induce proliferation in PCa cells. Activation of the MAPK phosphorylation cascade can be induced through non-genomic pathways of AR signaling and stimulate cell proliferation. Activated ERK can phosphorylate AR and its co-activators, and therefore, this feedback loop of the non-genomic AR signaling may induce genomic AR signaling in prostate cancer (58, 59). These are pathways that are well known in the literature pertaining to PCa; however, these classical genomic and non-genomic AR-signaling pathways are independent of the AR-tethering mechanism and the ability of AR to co-opt the ERK-docking sites in ELK1 to activate transcription, which are described in this report.

Overexpression of an ELK1-docking site mutant in which the ability to associate with AR was disrupted without disrupting the DNA binding domain had a dominant-negative effect on androgen-stimulated growth in PCa cells. This was unrelated to loss of ERK binding in the mutant ELK1 as the cells were insensitive to MEK inhibition. As the discrete DNA binding domain in the mutant ELK1 was not compromised, the mutant presumably still bound to ELK1 elements in the DNA; however, because the D-box was deleted in the mutant ELK1, our mapping data would predict that AR would not bind to it. The dominant-negative effect of the mutant ELK1 on androgen-stimulated growth is therefore consistent with this prediction. These observations provide an additional functional link between recruitment of AR by ELK1 and growth signaling by AR in PCa cells. The characterization of such discrete AR-interacting motifs should enable development of small molecule drugs that bind to those sites and selectively target dysregulated growth signaling in prostate cancer, while avoiding the global side effects of androgen ablation.

## Experimental Procedures

### 

#### 

##### Cell Culture and Reagents

LNCaP, CWR22Rv1 cells, and HeLa cell lines were from the American Type Culture Collection (Manassas, VA); 293FT cells were from Invitrogen. HeLa cells with a stably integrated minimal promoter-luciferase reporter containing five upstream Gal4 elements (*GAL4-TATA-LUC*) and expressing a Gal4-ELK1 fusion protein in which the Gal4 DNA binding domain was substituted for the ETS DNA binding domain of ELK1 were kindly provided by Dr. Johann Hofman (Innsbruck Medical University). These cells were then stably transduced with a vector expressing the full-length AR. Recombinant HeLa cells were also generated by stably transducing *GAL4-TATA-LUC* and a vector expressing the AR(A/B) domain fused to the VP16 transactivation domain (AR(A/B)-VP16). LNCaP cells were routinely grown at 37 °C in 5% CO_2_ in RPMI 1640 medium supplemented with 10% FBS (Invitrogen), 100 units/ml penicillin, 100 μg/ml streptomycin, 2 mm l-glutamine mixture (Invitrogen), and sodium pyruvate (1 mm) (Invitrogen). CWR22Rv1 cells were grown in phenol red-free RPMI 1640 medium supplemented with 10% FBS (Invitrogen), 100 units/ml penicillin, 100 μg/ml streptomycin, 2 mm l-glutamine mixture (Invitrogen). Parental and recombinant HeLa cells were grown in DMEM supplemented with 10% FBS and 100 units/ml penicillin, 100 μg/ml streptomycin, 2 mm l-glutamine mixture (Invitrogen). Additionally, the culture media for the recombinant HeLa cells included one or more of the following selection antibiotics: 100 μg/ml hygromycin (Invitrogen) (for Gal4-ELK1), 100 or 400 μg/ml geneticin (Invitrogen) (for *GAL4-TATA-LUC*), and 2 μg/ml puromycin (Sigma) (for AR or AR(A/B)-VP16). For hormone depletion, LNCaP cells were grown in phenol red-free RPMI 1640 medium supplemented with 10% heat-inactivated and charcoal-stripped FBS (Sigma) and 100 units/ml penicillin, 100 μg/ml streptomycin, 2 mm l-glutamine mixture for 96 h before each experiment. For hormone depletion, parental and recombinant HeLa cells were grown in phenol red-free DMEM supplemented with 5% heat-inactivated and charcoal-stripped FBS (Sigma) and 2 mm l-glutamine for 48 h before each experiment. Affinity-purified rabbit anti-human antibody to AR (sc-7305) and mouse antibodies to Gal4 (sc-510) and GAPDH (sc-47724) were purchased from Santa Cruz Biotechnology (Santa Cruz, CA). Rabbit monoclonal anti-human antibody to ERK1/ERK2 (ab17942), rabbit monoclonal anti-human to androgen receptor-ChIP grade antibody (ab74272), rabbit monoclonal anti-human antibody to ELK1 (ab32106), and mouse monoclonal anti-human antibodies to the androgen receptor (ab77557) and ELK1 (ab7712) were from Abcam (Cambridge, MA). R1881 was kindly provided by Dr. Steve Patrick (Wayne State University/Karmanos Cancer Institute, Detroit, MI). Testosterone was from Sigma. Lipofectamine^TM^ 2000 was purchased from Thermo Scientific (product number 78410). Trametinib was purchased from Selleckchem. AKTi-1/2 was purchased from EMD Millipore (Darmstadt, Germany). The endoribonuclease-prepared siRNAs were purchased from Sigma.

##### Purified Proteins

Full-length human AR expressed in insect cells and purified to >95% by affinity chromatography and FPLC (ab82609) was purchased from Abcam (Cambridge MA). Recombinant His-tagged ELK1 as well as His-tagged ELK1 mutated in both the D-box(Δ308–321) and the DEF motif (F397L, P398A) expressed from baculovirus-infected Sf9 cells were purified using nickel-agarose affinity chromatography. The proteins were eluted with 200 mm imidazole and dialyzed against 20 m HEPES, pH 7.9, containing 10% glycerol, 20 mm KCl, 2 mm MgCl_2_, 0.2 mm EDTA, 0.5 mm benzamidine, and 0.5 mm DTT. Purity of the proteins was estimated to be >85% by SDS-PAGE.

##### Surface Plasmon Resonance

Amine coupling kit, CM5 sensor chip, and HBS-N buffer (GE Healthcare) were used for SPR analysis. The rate and equilibrium binding constants of the interaction of AR with ELK1 or mutant ELK1 were determined using BIAcore 3000 (BIAcore, Piscataway, NJ). Affinity-purified AR polypeptide (ligand) was immobilized on a CM5 research grade sensor chip by an amine coupling method (60). The immobilization involved activation of carboxymethyl groups on a dextran-coated chip by reaction with *N*-hydroxysuccinimide, followed by covalent bonding of the ligand to the chip surface via amide linkages. Reference surfaces were prepared in the same manner but blocked with ethanolamine and thus contained no ligand. Kinetic binding analysis was carried out by injecting affinity-purified ELK1 polypeptide at different concentrations (0–160 nm) or mutant ELK1 (100 nm) into the flow cells (ligand and reference cell), and the interaction (response units (RU)) between analyte and ligand was recorded as the ligand RU minus the reference RU. Kinetic values were determined using BIAevaluation software (BIAcore), and the data were fitted with the model showing the closest match (61, 62). A 1:1 Langmuir binding model was generally selected, in which all the sensorgrams representing the different analyte concentrations were fitted simultaneously with the wide window of association and dissociation phases. Individual concentration curves were also evaluated to confirm the fitting data. The equilibrium dissociation constant (*K_d_*) was calculated by *K_d_* = *k*_off_/*k*_on_.

##### Plasmids

The expression plasmid for human full-length ELK1in the pCMV plasmid was purchased from OriGene (Rockville, MD). The pLenti-GIII-CMV-hELK1 lentiviral vector was from Applied Biological Materials Inc. The *GAL4-TATA-LUC* plasmid (pG5luc-Promega) and expression plasmid for VP16 and Gal4 were purchased from Promega (Madison, WI) (CheckMate Mammalian Two-hybrid System). The pRL plasmid encoding *Renilla* luciferase was purchased from Promega. The PatheDetect pFC-MEK1 trans-reporter plasmid with an S218E/S222E point mutation and internal deletion between amino acid residues 32 and 51 rendering it constitutively active was from Stratagene. The pLVX-AR-V7 plasmid and pLVX control plasmid were the kind gifts from Dr. Yan Dong, Tulane University (New Orleans, LA). Details of all other plasmid constructs generated in this work are provided in the supplemental material.

##### siRNA-mediated Gene Knockdown

The appropriate recombinant HeLa cells were plated in a 6-well plate (CytoOne, from USA Scientific) in DMEM supplemented with 10% FBS and 2 mm l-glutamine 24 h before transfection. The following day, cells were transfected with esiRNAs against ERK1 (MAPK3, 1 μg) and ERK2 (MAPK1, 1 μg) or 2 μg of control siRNA using Lipofectamine^TM^ 2000.

##### Co-immunoprecipitation

HeLa cells were transfected with the expression plasmid for AR and co-transfected with expression plasmid for WTELK1 or ELK1Δ308–321 or ELK1 FxLa. Cells were harvested in radioimmunoprecipitation assay lysis buffer and 1× protease inhibitor mixture 48 h post-transfection. Whole cell lysates (500 μg) were pre-cleared for 2 h using protein A-agarose beads (Calbiochem). Immunoprecipitation was performed by first incubating 100 μl of the protein A-agarose beads with 20 μg of the anti-rabbit androgen receptor antibody (ab74272) or negative control for 4 h. After washing the antibody-bound beads three times, 500 μg of the cell lysate was added and incubated at 4 °C overnight under rotary agitation. At the end of the incubation, the complexes were washed five times with the radioimmunoprecipitation assay buffer. The Western blotting was probed with mouse monoclonal AR antibody (ab77557) and mouse monoclonal ELK1 antibody (ab7712).

##### Other Experimental Methods

Transient transfection and luciferase reporter assays, Checkmate mammalian two-hybrid assay, lentivirus-mediated gene knockdown and gene expression, cell proliferation assay, Western blotting analysis, RNA isolation, reverse transcription, and real time PCR have been described in Ref. 31.

##### Statistical Analysis

All experiments were performed in triplicate and repeated at least three times. The error bars in all graphs represent the standard deviation. Statistical analysis was performed using one-way analysis of variance with post hoc and least square differences and/or *t* test. The *p* values are indicated in the figure legends.

## Author Contributions

R. R., P. E. S., and M. R., designed the study and wrote the paper. R. R., S. D., and T. M. performed and analyzed the experiments. R. R., M. P., V. C., and J. S. designed and constructed vectors for expression of mutant proteins. B. L. K. contributed database mining and related analysis. All authors reviewed the results and approved the final version of the manuscript.

## Supplementary Material

Supplemental Data

## Footnotes

2 The abbreviations used are:

AR: androgen receptor
PCa: prostate cancer
Gal4-DBD: Gal4-DNA binding domain
NR: nuclear receptor
SPR: surface plasmon resonance
ARE: androgen response element
CA: constitutively active
RU: response unit
SRF: serum-response factor
MTT: 3-(4,5-dimethylthiazol-2-yl)-2,5-diphenyltetrazolium bromide.
