# Supplementary material for: The Amino-terminal Domain of the Androgen Receptor Co-opts Extracellular Signal-regulated Kinase (ERK) Docking Sites in ELK1 Protein to Induce Sustained Gene Activation That Supports Prostate Cancer Cell Growth
Source: J Biol Chem. 2016 Oct 28;291(50):25983–98. doi: 10.1074/jbc.M116.745596 (PMC5207070; doi:10.1074/jbc.M116.745596)
Supplement: Supplemental Data [file supp_291_50_25983__index.html]

The Amino-terminal Domain of the Androgen Receptor Co-opts ERK Docking Sites in ELK1 to Induce Sustained Gene Activation that Supports Prostate Cancer Cell Growth — The Amino-terminal Domain of the Androgen Receptor Co-opts Extracellular Signal-regulated Kinase (ERK) Docking Sites in ELK1 Protein to Induce Sustained Gene Activation That Supports Prostate Cancer Cell Growth — Interactions of the Androgen Receptor and ELK1 — Supplemental Data 

# The Amino-terminal Domain of the Androgen Receptor Co-opts Extracellular Signal-regulated Kinase (ERK) Docking Sites in ELK1 Protein to Induce Sustained Gene Activation That Supports Prostate Cancer Cell Growth

## Supplemental Data

- Supplemental file 1 (.pdf, 162 KB) - Extension of preparation and design of plasmid constructs used in manuscript.
