# Supplementary material for: The Amino-terminal Domain of the Androgen Receptor Co-opts Extracellular Signal-regulated Kinase (ERK) Docking Sites in ELK1 Protein to Induce Sustained Gene Activation That Supports Prostate Cancer Cell Growth
Source: J Biol Chem. 2016 Oct 28;291(50):25983–98. doi: 10.1074/jbc.M116.745596 (PMC5207070; doi:10.1074/jbc.M116.745596)
Supplement: Supplemental Data [file 10.1074_M116.745596_jbc.M116.745596-1.pdf]

SUPPLEMENTAL MATERIAL

Additional Information on Plasmid Constructs

Construction of the $(ELK1)_2$-TATA-Luc plasmid and the ARE-TATA-Luc plasmid has been previously described (1). The Gal4-ELK1 fusion construct in which the DNA binding domain of ELK1 (amino acid residues 1-86) was deleted and replaced with the Gal4 DNA binding domain was constructed by PCR by using the ELK1-pCMV expression plasmid (Origene, Rockville, MD) as the template. The appropriate PCR primers were custom synthesized to generate the various amino-terminal and carboxyl-terminal deletion constructs of Gal4-ELK1 using the ELK1-pCMV expression plasmid as the PCR template and subcloned at *BamHI* (upstream) and *NotI* (downstream) sites in the pBind vector expressing Gal4 fusions. The following ELK1 deletions reported previously (2) i.e., pCMV5L ELK1 Δ31, pCMV5L ELK1 ΔD, pCMV5L ELK1 Δ32, pCMV5L ELK1 Δ32, pCMV5L ELK1 Δ24, pCMV5L ELK1 Δ19, pCMV5L ELK1 FxLa were subcloned into the appropriate vectors. PCR primers were designed to subclone each of the ELK1 deletions into the pBind vector at *BamHI* (upstream) and *NotI* (downstream) sites. All other ELK1 internal deletion and mutant constructs were generated using the The QuickChange II XL Site-Directed Mutagenesis kit (Agilent Technologies) according to the manufacturer's protocol. They include: Lentiviral ELK1 Δ308-321 and Lentiviral ELK1 FxLa, Gal4-ELK1Δ287-306, Gal4-ELK1Δ307-313, Gal4-ELK1Δ307-315, Gal4ELK1Δ400-407, Gal4-ELK1Δ331-340, and Gal4-ELK1Δ340-350.

The AR(A/B)-VP16 fusion construct was initially constructed using the VP16 expression plasmid from Promega. Using this plasmid as the PCR template, custom synthesized PCR primers were then used to amplify the AR(A/B)-VP16 sequence which was cloned into the

pCDH-CMV-MCS-EF1-Puro cDNA Cloning and Expression Vector (System Biosciences) at *NheI* (upstream) and *BamHI* (downstream) sites. The full length AR was subcloned from the pCMV expression vector (Origene) into the pCDH-CMV-MCS-EF1-Puro cDNA Cloning and Expression Vector (System Biosciences) at *NheI* (upstream) and *BamHI* (downstream) sites. Generation of the AR(A/B) expression plasmid in the pCDH vector has been described (1). Custom synthesized PCR primers were used to amplify and clone the five tandem Gal4 elements from the pG5luc vector into the pGreenFire1^{TM}-mCMV-EF1-Neo (Plasmid) at *SpeI* (upstream) and *BamHI* (downstream) sites.

All of the plasmid constructs generated above were sent to either the Plant-Microbe Genomics Facility for DNA Sequencing at The Ohio State University (Columbus, OH) or to Genewiz (South Plainfield, NJ) to verify DNA sequences before the constructs were used in the studies.

1. Patki, M., Chari, V., Sivakumaran, S., Gonit, M., Trumbly, R. and Ratnam, M. (2013) The ETS domain transcription factor ELK1 directs a critical component of growth signaling by the androgen receptor in prostate cancer cells. *The Journal of biological chemistry*, **288**, 11047-11065.
2. Evans, E.L., Saxton, J., Shelton, S.J., Begitt, A., Holliday, N.D., Hipskind, R.A. and Shaw, P.E. (2011) Dimer formation and conformational flexibility ensure cytoplasmic stability and nuclear accumulation of Elk-1. *Nucleic acids research*, **39**, 6390-6402.
